# Opportunistic and Organized Cervical Cancer Screening: Impact on Lesion Severity and Surgical Outcomes in 9830 Cervical Conizations

**DOI:** 10.3390/diagnostics16060839

**Published:** 2026-03-12

**Authors:** Mario Preti, Niccolò Gallio, Silvano Costa, Fulvio Borella, Paola Armaroli, Pedro Vieira-Baptista, Federica Zamagni, Federica Bevilacqua, Paola Garutti, Daniele Tota, Eleonora Robba, Ilaria Barbierato, Benedetta Pollano, Samuel Joseph Gardner-Medwin, Sara Babich, Camilla Cavallero, Ilaria Maschio, Alessio Mastrippolito, Alberto Revelli, Luca Marozio, Lauro Bucchi

**Affiliations:** 1 Gynecology and Obstetrics 1U, “AOU Città della Salute e della Scienza di Torino”, Department of Surgical Sciences, University of Turin, 10126 Turin, Italy; mario.preti@unito.it (M.P.); fulvio.borella87@gmail.com (F.B.); eleonora.robba@unito.it (E.R.); ilaria.barbierato@unito.it (I.B.); benedetta.pollano@unito.it (B.P.); samueljoseph.gardnermedwin@unito.it (S.J.G.-M.); sara.babich@unito.it (S.B.); camilla.cavallero@unito.it (C.C.); ilaria.maschio@unito.it (I.M.); alessio.mastrippolito@unito.it (A.M.); luca.marozio@unito.it (L.M.); 2 Gynecology and Obstetrics 2U, “AOU Città della Salute e della Scienza di Torino”, Department of Surgical Sciences, University of Turin, 10126 Turin, Italy; alberto.revelli@unito.it; 3 Department of Gynecology, Madre Fortunata Toniolo Hospital, Via Toscana 34, 40141 Bologna, Italy; costa.silvano@libero.it; 4 Epidemiology and Screening Unit, Reference Centre for Epidemiology and Cancer Prevention (CPO), Città della Salute e della Scienza University Hospital, Corso Bramante 88, 10126 Turin, Italy; paola.armaroli@cpo.it; 5 Hospital Lusíadas Porto, Avenida da Boavista 117, 4050-115 Porto, Portugal; pedrovieirabaptista@gmail.com; 6 Department of Gynecology-Obstetrics and Pediatrics, Faculdade de Medicina da Universidade do Porto, Alameda Prof. Hernâni Monteiro, 4200-319 Porto, Portugal; 7 Emilia-Romagna Cancer Registry, Romagna Cancer Institute, IRCCS Istituto Romagnolo per lo Studio dei Tumori (IRST) Dino Amadori, Via Piero Maroncelli 40, 47014 Meldola, Italy; federica.zamagni@irst.emr.it (F.Z.); lauro.bucchi@irst.emr.it (L.B.); 8 Gynecology and Obstetrics 3U, “AOU Città della Salute e della Scienza di Torino”, Department of Surgical Sciences, University of Turin, 10126 Turin, Italy; fede.bevi23@gmail.com; 9 Department of Obstetrics and Gynecology, University Hospital, 44124 Ferrara, Italy; pagarutti@gmail.com; 10 Pathology Unit, Città della Salute e della Scienza University Hospital, Corso Bramante 88, 10126 Turin, Italy; datota@cittadellasalute.to.it

**Keywords:** cervical intraepithelial neoplasia, cervical cancer screening, conization, health policy, cervical cancer, CIN3 linear extension

## Abstract

**Objectives**: To assess the impact of organized (OgS) versus opportunistic screening (OpS) on grade, extent, and surgical management of cervical lesions, and to evaluate human papillomavirus (HPV)-based versus cytology-based screening within OgS. **Methods**: This retrospective study analyzed 9830 women undergoing conization (1992–2021). Data included screening modality, histology, cervical intraepithelial neoplasia grade 3 (CIN3) linear extension, and cone volume. Statistical analysis employed chi-square test, Student’s *t*-tests, Cochran–Armitage test for trend, and Firth’s penalized multivariate logistic regression to identify independent predictors of invasive disease. **Results**: Of 9830 patients, 5097 (52%) were referred from OgS and 4733 (48%) from OpS. OgS patients were significantly older (40.0 vs. 37.0 years; *p* < 0.001). In the final decade, OgS achieved a significantly lower rate of invasive carcinomas compared to OpS (1.1% vs. 2.7%; *p* < 0.001). Mean CIN3 extension and cone volume were significantly lower in OgS (6.5 mm; 1150 mm^3^) than in OpS (7.1 mm; 1580 mm^3^; *p* < 0.001). Within OgS, HPV-detected CIN3 lesions were smaller than cytology-detected ones (5.9 vs. 6.4 mm; *p* < 0.001). Long-term analysis showed a borderline downward trend in invasive cancer for OgS (*p* = 0.089), whereas OpS remained stable at higher risk levels. Multivariate analysis confirmed the screening model as an independent predictor of invasiveness: OpS was associated with a two-fold increased risk of invasive cancer compared to OgS (adjusted odds ratio: 1.99; 95% confidence interval: 1.41–2.83; *p* < 0.001). **Conclusions**: OgS identifies high-grade precancers earlier and with smaller excisional requirements. OpS is associated with significantly higher invasive cancer rates and larger conizations. Multivariate data reinforce OgS as a superior framework, effectively halving the risk of invasive disease compared to OpS.

## 1. Introduction

Cervical cancer (CC) is the fourth most common cancer among women, and remains a significant global health challenge. Approximately 661,021 women were diagnosed with CC worldwide in 2022, and 348,189 died from the disease [1].

CC prevention has been a public-health success story in countries that adopted large-scale, effective screening, diagnosis, and treatment strategies. Indeed, the cornerstone of cervical cancer control is early detection of preinvasive lesions, which can be treated effectively to prevent progression to invasive carcinoma. Conization, in its various forms (cold knife, LASER, or loop electrosurgical excision procedure [LEEP]), remains the standard conservative surgical treatment for high-grade cervical intraepithelial neoplasia (CIN2–3) and selected glandular precursors [2,3], while conservative approaches such as thermal ablation have shown high efficacy, safety, and patient satisfaction in treating CIN1 with persistent high-risk human papillomavirus (HPV) infection, particularly in low-resource settings [3].

Historically, the Pap test offered the first major reduction in CC incidence and mortality [4]. In recent years, validated HPV primary testing, HPV vaccination, and self-sampling tools have reshaped screening paradigms and intensified the debate about the optimal mode of program delivery [5]. The distinction between organized (OgS) and opportunistic screening (OpS) matters because organizational design determines population coverage, test execution and follow-up, the extent to which benefits reach underserved groups, and limits over-testing and inefficiencies [6,7,8,9].

OgS programs typically targeting women aged 25–64/69 are characterized by defined intervals, standardized management algorithms, structured follow-up, and systematic quality assurance. Their implementation is resource-intensive but ensures equity, reduces variability, and improves long-term outcomes, including lower cervical cancer incidence and mortality.

By contrast, OpS relies on healthcare encounters outside a structured framework, initiated by physician recommendation during unrelated visits, self-referral, or in response to symptoms. Although often more flexible, OpS is inherently inequitable, disproportionately involving women of higher socio-economic status and paradoxically excluding those at greatest risk, especially those with lower socio-economic status instead. Moreover, it tends to rely predominantly on cytology, whereas OgS programs have increasingly transitioned to high-risk HPV testing as the primary screening tool, leveraging its superior sensitivity for detecting high-grade lesions [10,11,12].

We hypothesized that the context (OgS versus OpS) in which women are diagnosed with CIN influences the indications and outcomes of conization. Understanding how screening models influence conization practice is essential for optimizing resource allocation, minimizing overtreatment, and ensuring timely management of high-grade lesions.

The objective of this study is therefore to systematically compare conizations performed on women entering treatment through OgS versus OpS. By analyzing a large group of patients treated over three decades at a tertiary referral center, we aim to delineate the clinical, histopathologic, and surgical characteristics associated with each model. Ultimately, the study addresses a critical gap in the literature: how the structural organization of screening programs directly shapes not only CC epidemiology but also the clinical practice of conization. This study is part of a multiscope clinical study on a large cohort of patients undergoing cervical conization at the Sant’Anna Hospital of Obstetrics and Gynecology of Turin, Italy, a tertiary referral center serving the metropolitan area and nearby districts in Northern Italy.

## 2. Materials and Methods

### 2.1. Study Setting

With respect to cervical disease, the Sant’Anna Hospital of Obstetrics and Gynecology of Turin receives referrals both from women participating in the regional OgS as well as from OpS in private practice. This dual referral pathway enabled a comparative analysis of conization outcomes across distinct screening models.

### 2.2. Screening Procedures

The regional OgS program, targeting women aged 25–64 years, was launched in 1992. Up to March 2010, the protocol relied exclusively on a conventional Pap test every 3 years. Women whose cytology revealed atypical squamous cells of undetermined significance (ASC-US), including first-time findings, or more severe abnormalities were referred for colposcopic evaluation. Between 2010 and 2018, the program underwent a progressive transition from cytology-based screening to HPV testing for women aged 30–64 years. The Hybrid Capture 2 assay (Qiagen, Hilden, Germany) and the Anyplex II HPV Detection Kit (Seegene Diagnostics, Seoul, Republic of Korea) were employed.

Women testing HPV-positive were triaged by reflex cytology, and those with ASC-US or worse were referred to colposcopy. Co-testing was never adopted in this population.

**Figure 1:**
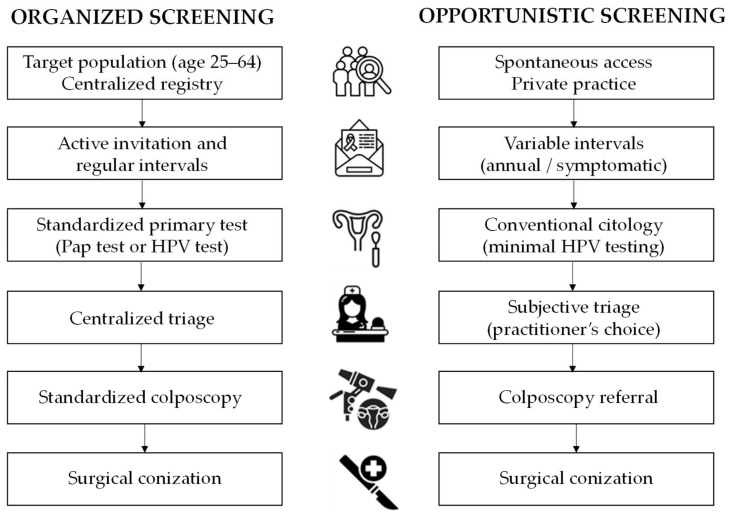
The flowchart illustrates the structural differences between the two screening pathways analyzed in the study. Organized screening: a population-based model characterized by centralized invitation, standardized primary testing (transitioning from Pap test to HPV-based screening), and centralized triage, ensuring a streamlined and monitored path to colposcopy. Opportunistic screening: spontaneous model based on private practice visits, characterized by variable testing intervals, frequent use of symptomatic testing, and subjective triage criteria. The structural advantages of the organized screening model lie in its evidence-based protocols, which aim to optimize surgical appropriateness and minimize low-risk interventions.

In the OpS setting, included in this analysis, cervical cytology was performed in private gynecologic offices, without any programmatic invitation system or centralized population registry linkage. The frequency of testing was not predetermined, and screening intervals varied widely among practitioners. In many cases, gynecologists repeated cytology annually during routine visits of asymptomatic women, even after previous negative results. In other instances, cytology was performed when women presented with vaginal symptoms, such as inflammatory or bloody discharge, rather than as part of a structured preventive strategy. Almost all smears were conventional slides rather than liquid-based preparations, and HPV testing was only occasionally performed in this context. This variability in testing practices reflects the lack of standardization inherent to OpS. A comparative flowchart of the screening pathways is available in Figure 1.

### 2.3. Colposcopic Assessment and Cervical Excision

Throughout the study period (1992–2021), the same colposcopic and excisional protocols were applied, regardless of the provenance (OgS or OpS).

Local excision was performed under local anesthesia using a monopolar electrosurgical generator. Wire-loop tungsten electrodes (0.2 mm thick) of 10 × 10 mm, 15 × 10 mm, 25 × 10 mm, or 25 × 15 mm were used depending on lesion size. For extensive lesions, needle electrodes were used to tailor the excision, and, since 2015, LASER excision has also been available. All procedures were performed by experienced colposcopists following institutional guidelines.

### 2.4. Histopathologic Processing and Reporting

**Figure 2:**
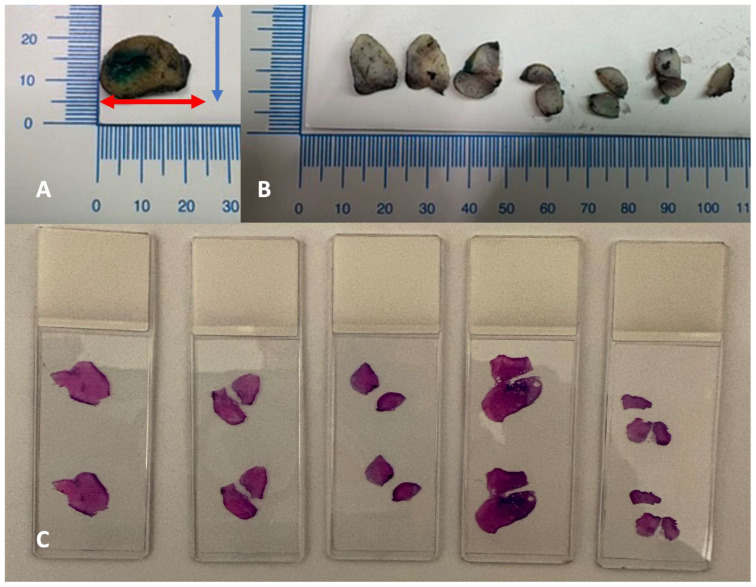
Processing protocol. (**A**) Every cervix conical excision is measured along three axes: longitudinal (red arrow), transverse (blue arrow), and length of cervical canal from external os to apex. Margins are inked with two different colors for the exocervical margin and the endocervical margin. (**B**) Sectioning into parallel sagittal slices, perpendicular to the external os, spaced 2–3 mm apart from the 9 to the 3 o’clock position. (**C**) For every section, a four-micrometer-thick section is stained with hematoxylin and eosin.

Resected tissue fragments after fixation in 10% neutral-buffered formalin were sectioned into parallel sagittal slices, spaced 2 mm apart from the 9 to the 3 o’clock position. Four-micrometer-thick sections were stained with hematoxylin and eosin (Figure 2).

During the study period, five pathologists evaluated the excised cones with double reading and the use of p16 for CIN2 diagnosis. The reports included:

- anteroposterior, transverse, and longitudinal (length) dimensions,
- histopathologic diagnosis (the most severe grade of disease identified in the specimen),
- linear extension of the lesion defined as the maximum linear measurement (in mm) of the high-grade lesion focus on the histologic slide,
- status of ectocervical, endocervical, and deep margins.

**Figure 3:**
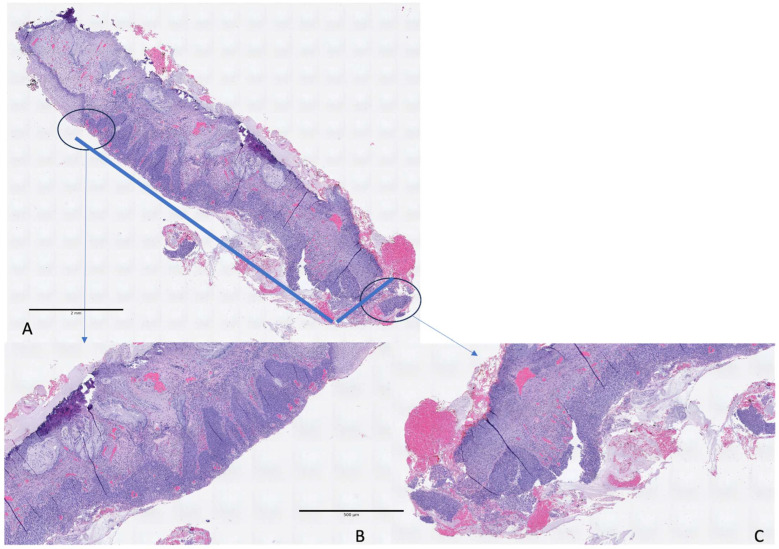
Eso-endocervical CIN3 with focal glandular involvement (**A**). Linear extension is measured from the exocervical (**B**) to the endocervical margin (**C**) (6.5 mm).

**Figure 4:**
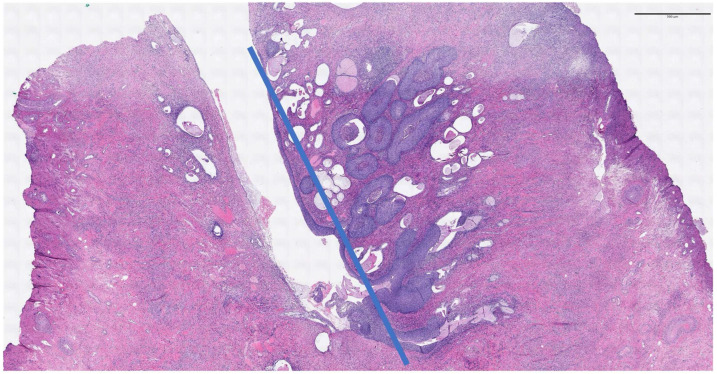
Endocervical CIN3 with massive glandular involvement. Linear extension is measured from the endocervix to the lower gland involved by intraepitelial lesion (6.7 mm).

Linear measurements are depicted in Figure 3 and Figure 4.

Measurements were performed in millimeters, using a micrometer eyepiece (10×) with a 10 mm scale divided into 100 intervals.

Conization volume was calculated based on the three diameters (anteroposterior, transverse, and longitudinal) provided by the pathologist after fixation, using a validated geometric formula for a semi-ellipsoid: π × [(anteroposterior diameter + transverse diameter)/4]^2^ × length [13].

**Figure 5:**
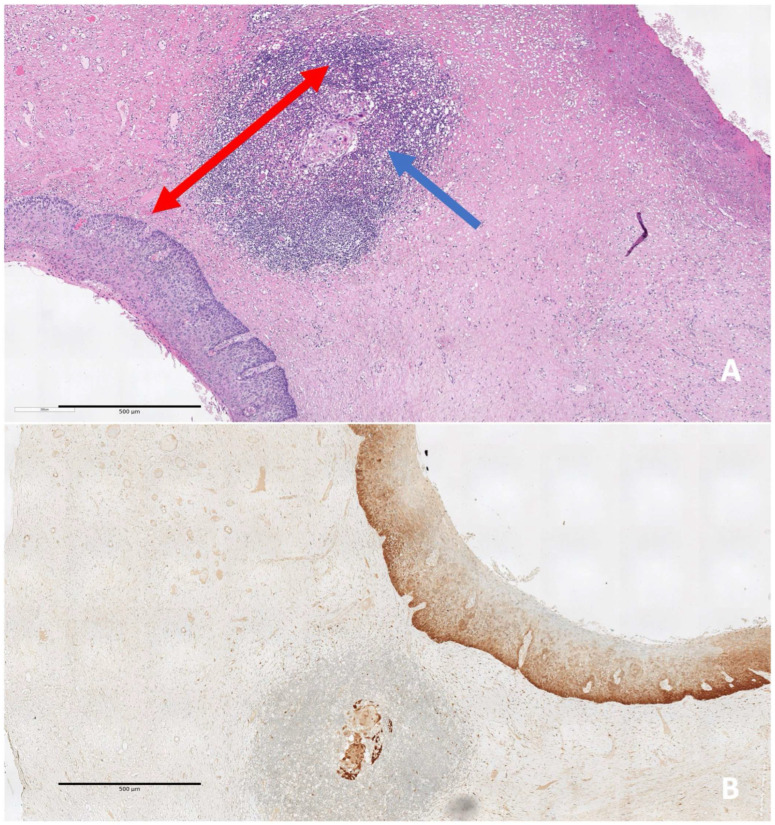
(**A**) CIN3 with microinvasive cancer (blue arrow). Depth of invasion is measured from the epithelial-stromal interface to the deepest point of stromal invasion (red arrow). (**B**) In this case, p16 immunostaining was performed to point out the focal stromal invasion.

Microinvasive CC was defined according to the FIGO classification as Stage IA1 (invasive carcinoma that can be diagnosed only by microscopy, with maximum depth of invasion < 5 mm) [14] (Figure 5).

AIS (adenocarcinoma in situ) was defined as a non-invasive high-grade glandular precursor limited to the cervical epithelium, without evidence of stromal invasion [15,16,17].

Invasive adenocarcinoma was defined as any malignant epithelial tumor of the cervix with glandular differentiation and evidence of stromal invasion, staged according to FIGO criteria [15].

### 2.5. Eligibility Criteria

Trained clinicians identified a consecutive group of 9830 women who underwent local cervical excision between 1992 and 2021. Clinical and histopathologic data were extracted using a standardized pro forma.

Inclusion criteria for this analysis were:

1. Age > 18 years
2. No previous treatment for intraepithelial/invasive cervical neoplasia
3. Cervical excision performed following abnormal cervical screening results
4. No prior cervical surgery or ablative treatment
5. Complete clinical and histopathologic records
6. No pregnancy at the time of conization

### 2.6. Statistical Analysis

Categorical variables were reported as counts and percentages, while continuous variables are presented as means and standard deviations (SD), ranges, and interquartile ranges (IQR). Differences between OgS and OpS were evaluated using Pearson’s chi-square test for categorical variables and Student’s *t*-test for continuous variables. For categorical variables with more than two groups, post hoc pairwise comparisons of proportions were performed following a significant global chi-square test, with Bonferroni correction applied to control for type I error inflation. The evolution of diagnostic performance over the 30-year study period was analyzed using the Cochran–Armitage test for trend. This test was specifically applied to assess the presence of a linear increase or decrease in the detection of high-risk lesion (CIN2, CIN3, AIS) and invasive cancer (micro-invasive squamous CC, invasive squamous CC, invasive adenocarcinoma) across the three decades (1992–2001, 2002–2011, and 2012–2021) for both screening models. Multivariate analysis was conducted using Firth’s penalized likelihood logistic regression to provide reliable estimates in the presence of rare events (invasive cases) and to prevent bias arising from quasi-complete separation of data, ensuring stable odds ratios (ORs) and confidence intervals (CIs) where standard maximum likelihood estimation might be unreliable. A two-sided *p* value < 0.05 was considered statistically significant. All statistical analyses were performed using R version 4.5.2.

## 3. Results

### 3.1. Study Population and Screening Model Comparison

**Figure 6:**
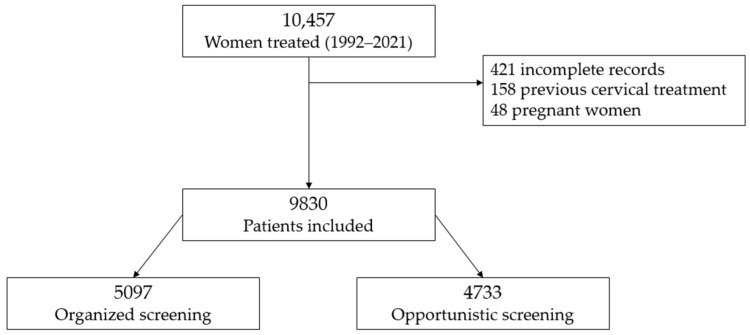
The flowchart illustrates the selection process of the study population.

**Figure 7:**
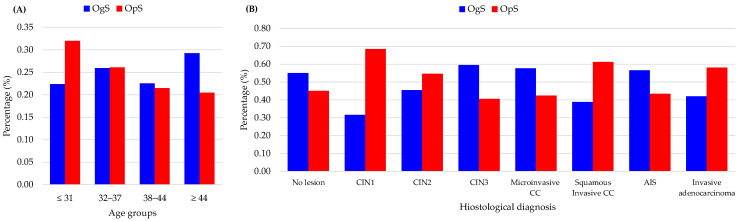
(**A**) Age distribution by screening model. (**B**) Histologic distribution by screening model.

A total of 9830 women were included in the analysis: 5097 were screened through the OgS and 4733 through OpS (Table 1, Figure 6). Participants in the OgS were significantly older than those referred from the OpS settings (mean age 40 ± 9.7 vs. 37 ± 10 years, *p* < 0.001). Age distribution across quartiles showed that OgS participation increased with age: from 43% in women ≤ 31 years to 61% in those ≥ 44 years (*p* < 0.001). Age distribution within the two screening populations confirmed that OgS was associated with an older age (Figure 7; panel (A)). 

Marked differences emerged in histopathologic outcomes (Table 1, Figure 7; panel (B)). Low-grade lesions (CIN1) were more frequent in the OpS than in the OgS group (68% and 32%, respectively), whereas high-grade CIN3 occurred predominantly within the OgS (60% vs. 40%, *p* < 0.001). Conversely, invasive squamous carcinomas were more frequent among OpS cases (61% vs. 39%, *p* < 0.001). The mean lesion extension was larger in the OpS group (7.1 ± 3.7 mm vs. 6.5 ± 3.1 mm, *p* < 0.001), as was the mean conization specimen volume (1442 mm^3^ vs. 1121 mm^3^, *p* < 0.001), suggesting later diagnosis and delay in management outside OpS.

### 3.2. Long-Term Analysis of Histologic Outcomes (1992–2021)

The diagnostic performance of the two screening models underwent a significant evolution over the 30-year study period (Table 2).

**Figure 8:**
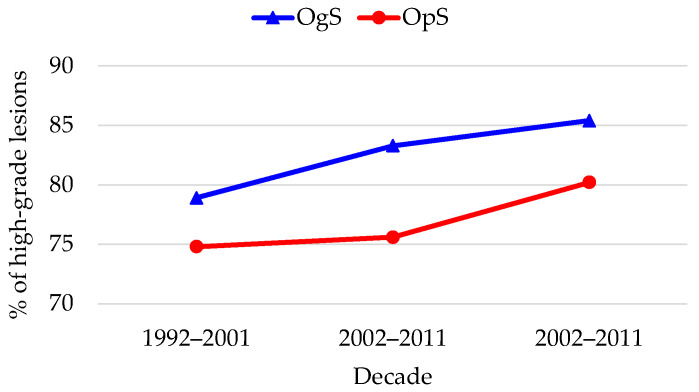
Trends in high-grade lesions (CIN2, CIN3, AIS) detection.

Initially, during the first decade (1992–2001), the OgS and OpS models showed baseline parity. No statistically significant differences were observed in the detection of CIN2-3/AIS (78.9% vs. 74.8%, respectively; *p* = 0.315) or negative specimen/CIN1 (19.1% vs. 23.2%), suggesting comparable diagnostic yields at the start of the observation (Figure 8).

A significant divergence emerged in the second decade (2002–2011). The OgS model demonstrated a superior ability to intercept high-grade precursors (CIN2, CIN3, AIS) with detection rising to 83.3%, significantly outperforming the OpS model, which remained stagnant at 75.6% (*p* < 0.001). This shift was accompanied by a significant reduction in low-risk histologic findings within the OgS group (15.2% vs. 22.6% in OpS), indicating improved triage and surgical appropriateness. This trend was consolidated in the most recent decade (2012–2021), where the OgS model maintained an elevated high-risk detection rate (85.4%) and achieved its lowest rate of low-risk detections (13.5%), significantly lower than in the OpS group (17.1%; *p* < 0.001), characterized by a higher proportion of invasive cases (*p* < 0.001).

Linear trend analysis (Cochran–Armitage test) further clarified these patterns (Table 3). For the OgS, high-risk lesion detection remained consistently high and stable across the 30 years (Z = −1.176, *p* = 0.239 for trend), while the OpS showed an increase in high-risk precursors detection (Z = −2.007, *p* = 0.045).

**Figure 9:**
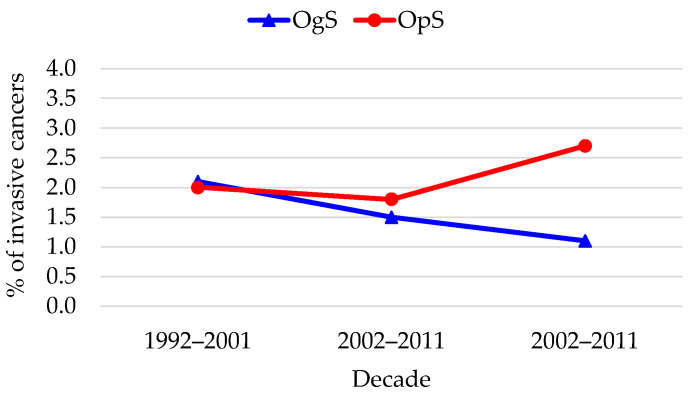
Trends in invasive cancer (invasive squamous CC, invasive adenocarcinoma) detection.

Regarding invasive cancer, the two models showed diametrically opposite directions (Figure 9). In the OgS group, proportions of pure invasive carcinoma significantly decreased from 2.1% to 1.1%, while the OpS group saw an increase from 2.0% to 2.7%. These trends were further analyzed (Table 4). The organized model showed a borderline downward trend (Z = 1.669; *p* = 0.089), suggesting an increasingly protective effect, while the opportunistic model demonstrated no improvement, with a non-significant stable-to-upward progression (Z = −0.682; *p* = 0.495).

### 3.3. Impact of Patient Age on Lesion Severity

To evaluate whether the observed differences in disease severity were influenced by demographic factors, an age-stratified analysis was performed (Table 5). Results showed that women aged ≤ 39 years accounted for 66–70% of CIN1–2 and 58% of CIN3, while invasive carcinoma was more common among women > 39 years (64%, *p* < 0.001). When divided into quartiles (Table 6), the youngest group (≤31 years) represented 35% of CIN1 and CIN2, whereas microinvasive and invasive cancers rose progressively with age, reaching a peak at 38% and 47%, respectively, in women > 45 years. 

### 3.4. Subgroup Analysis Within the OgS: Pap Test vs. HPV Testing

Finally, to assess the impact of the shift to primary HPV-based screening within the organized framework, we compared Pap test–based and HPV-based screening modalities exclusively in the OgS population (Table 7). This comparison revealed the age shift. The mean age was 37 ± 8.9 years for Pap testing and 43 ± 8.7 years for HPV testing (*p* < 0.001). HPV testing predominated in women ≥ 44 years (62%) and was associated with smaller CIN3 extension (5.9 ± 3.7 mm vs. 6.4 ± 3.1 mm, *p* < 0.001). Although both modalities detected similar proportions of CIN3, adenocarcinoma in situ was identified more frequently with HPV screening (56% vs. 44%), indicating higher sensitivity for glandular lesions.

### 3.5. Multivariate Analysis of Risk Factors for Invasive Disease

A multivariate logistic regression using Firth’s penalized method was performed to identify independent predictors of invasive carcinoma, excluding microinvasive cases (Table 8). After adjusting for age and time period, the screening model emerged as a highly significant independent predictor of invasive disease. Patients in the OpS group had a two-fold increased risk of presenting with invasive cancer compared to those in the OgS group (adjusted OR [aOR]: 1.99; 95% CI: 1.41–2.83; *p* < 0.001).

Age was also significantly associated with invasiveness (aOR: 1.06; 95% CI: 1.04–1.07; *p* < 0.001). Conversely, the study period (2012–2021 vs. 2002–2011) showed no significant independent impact on the probability of invasive cancer (aOR: 1.03; 95% CI: 0.73-1.46; *p* = 0.845). These findings suggest that while the biological risk increases with age, the organized screening model provides a superior protective effect against the progression to or detection of pure invasive carcinoma regardless of the time period.

## 4. Discussion

According to the EU Advisory Committee on Cancer Prevention, cancer screening in Europe should be offered only in OgS programs with quality assurance at all levels [18,19]. Indeed, CC screening can reduce disease incidence up to 80% if quality is optimal at every step in the screening process, where guidelines must be followed throughout the procedure [20]. OgS has an explicit target population (denominator), active invitation and recall, central registries, standardized screening intervals and tests, quality assurance for laboratories and clinicians, and systems for tracking follow-up and outcome evaluation.

OpS remains widely practiced in private gynecologic care, where many women continue to seek evaluation either as part of routine check-ups or in response to subjective symptoms or personal concerns. OpS lacks centralized invitations or registries. Testing occurs at the initiative of clinicians or patients and is heterogeneous in interval, test quality, and follow-up pathways [6,7,8,9,21,22]. OpS reflects both the longstanding habits of gynecologists and women, and the perceived reassurance associated with individualized care, despite the well-documented advantages of population-based OgS programs. Thus, it continues to play a complementary yet less regulated role, particularly in settings where cultural, logistical and organizational factors limit adherence to structured national screening pathways.

The structural differences between OgS and OpS produce substantially different outcomes and, in our patients, these can be linked to clear demographic and histopathologic differences. These differences appear to mirror both the distinct organizational frameworks of the two screening models and the biological course of HPV-related disease, which progresses differently depending on age, screening frequency, and diagnostic timing.

Women participating in OgS were older on average than those in OpS (40 ± 9.7 vs. 37 ± 10 years, *p* < 0.001), a difference that persisted across quartiles, indicating reaching a larger proportion of middle-aged women, whereas OpS disproportionately involved younger individuals. These findings illustrate the systemic and biological consequences of differing screening paradigms and reinforce the superiority of structured, population-based approaches in achieving balanced detection across disease stages. OpS predominated among women aged ≤ 31 years (57%), whereas OgS captured a higher proportion of women ≥ 44 years (61%). This age gradient suggests that opportunistic testing is driven largely by reproductive-age attendance and individual initiative, often for contraceptive, fertility, or sexual health reasons, whereas OgS achieves broader engagement both among women in peri- and post-reproductive age through systematic invitation. Consequently, the OgS better aligns with the epidemiologic window of highest risk for progression from HPV persistence to high-grade disease.

The shift from OpS to OgS, particularly when centered on HPV primary testing, serves as a critical safeguard against the over-treatment of young patients. While OpS often results in the frequent detection of transient, low-grade lesions in low-risk young women—leading to a cascade of unnecessary colposcopies and excisional procedures—organized programs utilize evidence-based age thresholds and extended screening intervals to allow for the natural immunological clearance of HPV. By leveraging the high negative predictive value of HPV testing and implementing rigorous triage protocols (e.g., reflex cytology for HPV-positive cases), OpS effectively distinguishes between transient infections and persistent high-grade disease. This ‘watchful waiting’ approach significantly reduces the incidence of surgical interventions, such as LEEP or cold-knife conization, thereby minimizing the associated long-term obstetric risks, including cervical insufficiency and preterm birth, without compromising the early detection of invasive carcinoma.

Histology differs sharply between screening modalities. OpS yielded a predominance of low-grade lesions (68% of CIN1 and 55% of CIN2 cases), whereas OgS detected proportionally more CIN3 (60% vs. 40%, *p* < 0.001). This pattern likely reflects the different screening intervals, follow-up protocols, and adherence levels. Opportunistic testing tends to identify transient HPV-related abnormalities in younger women, often before progression, while, in contrast, OgS, by systematically recalling women at defined intervals, may detect more advanced intraepithelial neoplasia accumulated over time. In OpS, where women often undergo repeated testing at short and biologically unjustified intervals, a patient is likely to have a sense of disease persistence or progression, independent of the actual natural history of HPV infection. Consequently, she may actively request treatment or be persuaded to undergo excisional procedures, even when clinical guidelines would recommend continued surveillance. In contrast, women managed within OgS programs are protected by standardized algorithms and evidence-based timing, which help to prevent unnecessary interventions and the psychological burden associated with overtreatment.

The analysis of our 30-year group of patients provides a unique perspective on the maturation of screening programs. While OgS and OpS showed baseline parity in the first decade (1992–2001), a significant divergence emerged from the second decade onwards. Our data demonstrate that the OgS model achieved a superior and stable ability to intercept high-grade precursors, reaching its peak performance in the most recent decade (85.4% detection rate). In contrast, the OpS model showed a late-onset improvement in high-grade detection, which was paradoxically accompanied by a rising trend in invasive cases, suggesting a delayed alignment with evidence-based practices. Crucially, our multivariate analysis redefines these findings. When adjusted for temporal trends and age, the screening model emerged as an independent predictor of invasive disease. Specifically, women in the OpS faced a two-fold higher risk of being diagnosed with pure invasive cancer compared to those in the organized program (aOR 1.99; 95% CI: 1.41–2.83, *p* < 0.001). This indicates that the observed statistical divergence between the two models in the last 20 years is driven not only by the structural efficiency of the OgS in reducing low-risk histologic findings, but also by its superior capacity to prevent the progression to—or ensure the earlier detection of—invasive carcinoma. The stability of the OgS performance over three decades, compared to the concerning trend of the OpS, reinforces the role of organized programs as the most reliable framework for long-term CC control.

Interestingly, invasive cancers were proportionally more common in the OpS group (with the proportion of invasive cases rising to 2.7% in the final decade vs. 1.1% in OgS), suggesting that despite identifying many low-grade lesions, opportunistic testing fails to ensure early interception of some progressive cases. The borderline downward trend in invasive cancer detection observed within the OgS group (*p* = 0.089), which was notably absent in the OpS group, highlights the potential of organized screening to reduce cancer incidence—a protective signal that is missing when testing depends solely on individual initiative or physician discretion.

Lesion size and conization metrics also differed significantly between the two settings. The mean linear extension of CIN3 lesions was smaller among women identified through OgS (6.5 mm) compared with those diagnosed in OpS (7.1 mm; *p* < 0.001). This difference, though apparently modest, is clinically meaningful, as it suggests that OgS detects lesions at an earlier phase of intraepithelial expansion, before they reach a wider surface involvement. In parallel, the mean conization volume was significantly lower in the OgS group (1121 mm^3^ vs. 1442 mm^3^; *p* < 0.001), consistent with more conservative excisional procedures and greater adherence to standardized surgical criteria.

When stratified by age, the data revealed a coherent natural history of HPV-induced disease. Younger women (≤39 years) exhibited the highest prevalence of CIN1 and CIN2 (66–69%), whereas invasive lesions clustered in older groups (64%). In the extended quartile analysis, women ≤ 31 years contributed disproportionately to CIN1 (35%) and CIN2 (35%), but only 5–10% of invasive carcinomas, whereas those >45 years accounted for 47% of invasive cases. This age-related gradient mirrors the biological latency between viral infection, persistence, and transformation, and underscores the importance of maintaining surveillance into midlife.

In OgS, comparison of Pap test-based and HPV test-based modalities revealed further distinctions. Pap test-based testing identified proportionally more CIN2 and CIN3 lesions (63% and 55%, respectively) than HPV screening (37% and 45%), while HPV testing was associated with a higher proportion of AIS and invasive adenocarcinoma (56% vs. 44%). This pattern underscores HPV testing’s superior sensitivity for early glandular and endocervical lesions, which are often missed by cytology. Moreover, the mean extension of CIN3 lesions was smaller in women identified through HPV-based screening (5.9 mm vs. 6.4 mm, *p* < 0.001), indicating that HPV testing tends to detect lesions at an earlier stage in their development. These findings align with recent evidence demonstrating that HPV-detected CIN3 lesions are not only smaller in linear extension but also associated with a 50% lower risk of stromal microinvasion [23]. This protective effect is likely mediated by a lower degree of glandular crypt involvement, as recently reported in large-scale studies. This earlier identification allows for less extensive excisions and, consequently, a more conservative surgical approach with critical implications for reproductive health, as cone volume is a well-established predictor of adverse obstetric outcomes, including preterm birth, cervical insufficiency, and neonatal morbidity. By reducing overtreatment and limiting the extent of excision, OgS aligns cancer prevention with preservation of reproductive potential, a dual imperative in modern gynecologic oncology.

These findings further strengthen the rationale for the transition from cytology-based to HPV-based primary screening, as HPV testing not only improves the timing and precision of detection but also contributes to reducing unnecessary tissue removal and treatment morbidity. The work of Paulauskiene and colleagues in Lithuania highlighted how the transition to organized HPV-based screening significantly reshapes the clinical landscape, reducing both bigger conizations and the incidence of advanced disease [6]. Similarly, Teixeira et al. have shown that women entering treatment through OgS more often present with CIN3 or microinvasive squamous CC, while OpS women contribute disproportionately to the pool of invasive cancers and unnecessary conizations for CIN1 [10]. It is possible to surmise from these evaluations that OgS consistently achieves higher and more equitable coverage, as well as more reliable reductions in incidence than OpS [6,22,24,25]. Also, classic cost-effectiveness modeling and empirical comparisons have repeatedly shown opportunistic cytology to be more expensive and less efficient than OgS, employing evidence-based intervals [9,19,20,26,27,28,29,30,31,32].

Data from national screening programs indicate that OgS strategies substantially improve participation rates, particularly in countries with higher levels of gender inequality. As shown by Willems and Paulauskiene [6,33], when systematic invitation and recall mechanisms are in place, women’s adherence to cervical screening increases markedly, overcoming barriers linked to education, autonomy, and access to healthcare. Instead, in OpS, participation depends largely on individual initiative and financial means, reinforcing social disparities. D’Souza and colleagues highlighted this imbalance in low-resource settings, such as India, where only 22% of women aged 15–45 years have ever undergone a cervical examination, and mostly in private facilities [34]. Thus, OgS acts as both a public health intervention and a social equalizer. Studies confirm that organized HPV-based screening reduces the need for extensive treatment, lowering the risk of advanced disease [35]. Additionally, cost-effectiveness analyses consistently show that opportunistic cytology is both more expensive and less effective than organized HPV-based screening [35].

Our analysis has some limitations. First, the retrospective design carries inherent risks of selection bias and incomplete data capture. Although the large sample size and integration with regional prevention registries strengthen reliability, some residual confounding may persist. Second, while we stratified outcomes by screening modality, individual-level adherence to OgS was variable, and cross-over between systems may have occurred. In the third place, the design of the study needs to be critically considered. Ideally, the results of OgS activities should be compared with those of the OpS practice using absolute rates rather than patient proportions and distributions by disease characteristics. In fact, the almost complete lack of data from first-level screening in the OpS setting makes it virtually impossible to use this approach. Fourth, the retrospective design prevented the inclusion of certain baseline characteristics, such as body mass index, education level, smoking habit, socio-economic status, and presence of symptoms, which were not systematically recorded over the three-decade study period. These factors are known to influence health-seeking behavior and may contribute to the differences observed between organized and opportunistic screening participants. Finally, obstetric outcomes and vaccination status were not available for this group of patients, limiting direct evaluation of the reproductive consequences associated with cone volume, a critical area for future investigation despite its well-established relevance in the literature [19].

In mixed systems such as Italy, strategies to progressively phase out OpS should be considered, while ensuring continuity of care for women currently outside OgS. Policy recommendations should prioritize organized, population-based programs and discourage opportunistic over-testing through provider education, incentive realignment, and minimum reporting requirements for all cervical tests [7,8,9,25,36,37,38]. In parallel, transitional models should ensure that women accustomed to opportunistic care are not lost to follow-up, especially in underserved or mobile populations.

## 5. Conclusions

Taken together, the data presented in our study reinforce the structural advantages of OgS systems. Such programs ensure standardized intervals and quality-assured triage pathways, which collectively yield a higher proportion of clinically significant precancers detected before invasion and allow for smaller excisional volumes. OpS, in contrast, captures a younger population at lower oncologic risk yet leads to a higher frequency of unnecessary interventions for transient lesions, as demonstrated by our multivariate model, a significantly higher risk of invasive cancer due to gaps in continuity. Our analysis over three decades confirms that while the OgS has achieved a progressive reduction in the proportion of invasive carcinomas (dropping to 1.1%), the OpS has seen these diagnoses increase, reaching 2.7% in the most recent period. From a public health perspective, this dichotomy underscores the inefficiency of relying on opportunistic testing to achieve population-level cancer control.

Furthermore, the integration of HPV primary testing within OgS achieves superior risk stratification, balanced age coverage, and more conservative treatment profiles, whereas OpS, despite its accessibility, remains inherently fragmented and less effective in cancer prevention. Our multivariate analysis further strengthens these findings.

The data advocate for the continued expansion of organized, population-based screening strategies as the cornerstone of cervical cancer control in the post-HPV vaccination era. Our findings emphasize that organized HPV-based screening not only improves detection efficiency but also minimizes surgical morbidity, reinforcing its role as a measure of healthcare equity and quality.

## Figures and Tables

**Table 1:** Clinical and pathologic features and outcomes according to the type of screening (OgS vs. OpS).

	Total Patients (N = 9830)	OgS (N = 5097)	OpS (N = 4733)	*p*
Age (mean as cut-off)				
≤39	5953	2841 (48%)	3112 (52%)	<0.001 *
>39	3877	2256 (58%)	1621 (42%)	
Age (IQR)				
≤31	2654	1139 (43%) _a_	1515 (57%) _b_	<0.001 *
32–37	2555	1321 (52%) _a_	1234 (48%) _a_	
38–44	2163	1147 (53%) _a_	1016 (47%) _a_	
≥44	2458	1490 (61%) _a_	968 (39%) _b_	
Histology				
No lesion	706	388 (55%) _a_	318 (45%) _a_	<0.001 *
CIN1	944	298 (32%) _a_	646 (68%) _b_	
CIN2	2957	1343 (45%) _a_	1614 (55%) _b_	
CIN3	4823	2868 (60%) _a_	1955 (40%) _b_	
Microinvasive CC	118	68 (58%) _a_	50 (42%) _a_	
Squamous Invasive CC	129	50 (39%) _a_	79 (61%) _b_	
AIS	122	69 (57%) _a_	53 (43%) _a_	
Invasive adenocarcinoma	31	13 (42%) _a_	18 (58%) _a_	
CIN3 extension in mm (mean ± SD, range)	6.7 ± 3.3, 1–34	6.5 ± 3.1, 1–25	7.1 ± 3.7, 1–35	<0.001 °
Conization volume in mm^3^ (mean ± SD, range)	1270 ± 635, 100–4680	1150 ± 575, 100–4500	1580 ± 790, 180–4680	<0.001 °
Conization volume in mm^3^ (mean as cut-off)				
≤1270	6623	3669 (55%)	2954 (45%)	0.001 *
>1270	2495	1163 (47%)	1332 (53%)	

Abbreviations: OgS: organized screening; OpS: opportunistic screening; IQR: interquartile range; CIN: cervical intraepithelial neoplasia; CC: cervical carcinoma; AIS: adenocarcinoma in situ; SD: standard deviation. _a,b_ letters denote significant pairwise differences (Bonferroni-corrected) within each row. * ***p*** value obtained with chi-square test; ° ***p*** value obtained with ***t***-student test.

**Table 2:** Distribution of cervical lesions by screening model and decade.

Decade	Screening Model	Negative/CIN1	CIN2, CIN3, AIS	Invasive Carcinoma	*p*
1992–2001	OgS	37 (19%) _a_	153 (78.9%) _a_	4 (2.1%) _a_	0.315
	OpS	249 (23.2%) _a_	907 (74.8%) _a_	21 (2.0%) _a_	
2002–2011	OgS	186 (15.2%) _a_	1009 (83.3%) _a_	18 (1.5%) _a_	<0.001 *
	OpS	428 (22.6%) _b_	1639 (75.6%) _b_	38 (1.8%) _b_	
2012–2021	OgS	503 (13.5%) _a_	3030 (85.4%) _a_	39 (1.1%) _a_	<0.0001 *
	OpS	247 (17.1%) _b_	1164 (80.2%) _b_	40 (2.7%) _b_	

Abbreviations: CIN: cervical intraepithelial neoplasia; AIS: adenocarcinoma in situ; OgS: organized screening; OpS: opportunistic screening. Invasive carcinoma includes invasive squamous carcinoma and invasive adenocarcinoma. _a,b_ letters denote significant pairwise differences (Bonferroni-corrected). * *p* value obtained with chi-square test.

**Table 3:** Cochran–Armitage test for trend in high-risk (CIN2, CIN3, AIS) lesion detection (1992–2021).

Screening Model	Trend Description	Z-Score	*p*
Organized	Stable High-Performance	−1.176	0.239
Opportunistic	Gradual Increase	−2.007	0.045

**Table 4:** Cochran–Armitage test for trend in invasive cancer detection (1992–2021).

Screening Model	Trend Description	Z-Score	*p*
Organized	Borderline decrease	1.669	0.089
Opportunistic	Stable	−0682	0.495

**Table 5:** Histopathologic features and outcomes according to age groups (defined based on the mean age).

	Total Patients	Age ≤ 39	Age > 39	*p*
Histology				
No lesion	706	314 (45%) _a_	392 (55%) _b_	<0.001 *
CIN1	944	618 (66%) _a_	326 (34%) _b_	
CIN2	2957	2044 (70%) _a_	880 (30%) _b_	
CIN3	4823	2799 (58%) _a_	2024 (42%) _b_	
Squamous Microinvasive CC	118	55 (47%) _a_	63 (53%) _b_	
Squamous Invasive CC	129	47 (36%) _a_	82 (64%) _b_	
AIS	122	64 (53%) _a_	58 (47%) _a_	
Invasive adenocarcinoma	31	17 (55%) _a_	14 (45%) _a_	

Abbreviations: CIN: cervical intraepithelial neoplasia; CC: cervical carcinoma; AIS: adenocarcinoma in situ. _a,b_ letters denote significant pairwise differences (Bonferroni-corrected) within each row. * *p* value obtained with chi-square test.

**Table 6:** Histopathologic features and outcomes according to age groups (defined based on the interquartile range).

	Total Patients	Age ≤ 31	Age 32–37	Age 38–44	Age > 45	*p*
Histology						
No lesion	706	143 (20%) _a_	132 (19%) _a_	146 (21%) _a_	285 (40%) _b_	<0.001 *
CIN1	944	334 (35%) _a_	214 (23%) _b_	186 (20%) _b_	210 (22%) _b_	
CIN2	2957	1015 (34%) _a_	805 (27%) _b_	618 (21%) _b_	519 (18%) _c_	
CIN3	4823	1112 (23%) _a_	1311 (27%) _b_	1104 (23%) _a_	1296 (27%) _b_	
Squamous Microinvasive CC	118	6 (5%) _a_	38 (32%) _b_	29 (25%) _b_	45 (38%) _b_	
Squamous Invasive CC	129	13 (10%) _a_	19 (15%) _a_	36 (28%) _b_	61 (47%) _b_	
AIS	122	26 (21%) _a_	28 (23%) _a_	32 (26%) _a_	36 (30%) _a_	
Invasive adenocarcinoma	31	6 (19%) _a_	8 (26%) _a_	11 (36%) _a_	6 (19%) _a_	

Abbreviations: CIN: cervical intraepithelial neoplasia; CC: cervical carcinoma; AIS: adenocarcinoma in situ. _a,b,c_ letters denote significant pairwise differences (Bonferroni-corrected) within each row. * *p* value obtained with chi-square test.

**Table 7:** Clinical and histopathologic features according to screening method (Pap test vs. HPV test) evaluated in OgS.

	Pap Test	HPV Test	*p*
Age (mean ± SD, range)	37 ± 8.9, 24–74	43 ± 8.7, 18–79	<0.001 °
Age (mean)			
≤39	1933 (68%)	908 (32%)	<0.001 *
>39	998 (44%)	1258 (56%)	
Age (IQR)			
≤31	1001 (88%)_a_	138 (12%) _b_	<0.001 *
32–37	745 (56%) _a_	576 (44%) _a_	
38–44	551 (48%) _a_	596 (52%) _a_	
≥44	565 (38%) _a_	925 (62%) _b_	
Histology			
No lesion	206 (53%) _a_	182 (47%) _a_	<0.001 *
CIN1	187 (63%) _a_	111 (37%) _a_	
CIN2	852 (63%) _a_	491 (37%) _b_	
CIN3	1576 (55%) _a_	1292 (45%) _b_	
Squamous Microinvasive CC	46 (68%) _a_	22 (32%) _a_	
Squamous Invasive CC	32 (64%) _a_	18 (36%) _a_	
AIS	30 (44%) _a_	39 (56%) _b_	
Invasive adenocarcinoma	13 (54%) _a_	18 (46%) _a_	
CIN3 extension in mm (mean ± SD, range)	6.4 ± 3.1, 1–35	5.9 ± 3.7, 1–20	<0.001 °

Abbreviations: CIN: cervical intraepithelial neoplasia; IQR: interquartile range; CC: cervical carcinoma; AIS: adenocarcinoma in situ; SD: standard deviation. _a,b_ letters denote significant pairwise differences (Bonferroni-corrected) within each row. * *p* value obtained with chi-square test; ° *p* value obtained with *t*-student test.

**Table 8:** Multivariate logistic regression (Firth’s method) for factors associated with invasive cancer.

Comparison	aOR	CI	*p*
Screening (OpS vs. OgS)	1.99	1.41–2.83	<0.001
Age (continuous)	1.06	1.04–1.07	<0.001
2002–2011 vs. 1992–2001	0.81	0.44–1.48	0.491
2012–2021 vs. 1992–2001	0.84	0.43–1.63	0.612
2012–2021 vs. 2002–2011	1.03	0.73–1.46	0.845

Abbreviations: aOR: adjusted odds ratio; CI: confidence interval; OgS: organized screening; OpS: opportunistic screening.

## Data Availability

The original contributions presented in this study are included in the article. Further inquiries can be directed to the corresponding author.
